# Supplementary material for: Evaluation of transcutaneous electrical acupoint stimulation for improving pain and cognitive function in elderly patients around the perioperative period of hip replacement surgery: A meta-analysis
Source: PLoS One. 2024 Oct 21;19(10):e0309673. doi: 10.1371/journal.pone.0309673 (PMC11493289; doi:10.1371/journal.pone.0309673)
Supplement: S1 File — (DOCX) [file pone.0309673.s002.docx]

| No. | Author, Year | Study Type | Included/Excluded | Reason for Exclusion (if applicable) |
| --- | --- | --- | --- | --- |
| 1 | Sun K et al. 2018[1] | Randomized Controlled Trial | Included | Not applicable |
| 2 | Yang et al. 2023[2] | Randomized Controlled Trial | Included | Not applicable |
| 3 | Lan et al. 2017[3] | Randomized Controlled Trial | Included | Not applicable |
| 4 | Sun PH et al.2019[4] | Randomized Controlled Trial | Included | Not applicable |
| 5 | Duan et al. 2019[5] | Randomized Controlled Trial | Included | Not applicable |
| 6 | Yin et al. 2015[6] | Randomized Controlled Trial | Included | Not applicable |
| 7 | Lu et al. 2019[7] | Randomized Controlled Trial | Included | Not applicable |
| 8 | Liu et al. 2019[8] | Randomized Controlled Trial | Included | Not applicable |
| 9 | Peng ta al. 2019[9] | Randomized Controlled Trial | Included | Not applicable |
| 10 | Wang DD et al. 2016[10] | Randomized Controlled Trial | Included | Not applicable |
| 11 | Wang JW et al. 2017[11] | Randomized Controlled Trial | Included | Not applicable |
| 12 | Ge et al. 2023[12] | Randomized Controlled Trial | Included | Not applicable |
| 13 | Li et al. 2020[13] | Randomized Controlled Trial | Included | Not applicable |
| 14 | Tan et al.2024[14] | Meta-analysis | Excluded | Non-randomized controlled trial |
| 15 | Guo et al.2023[15] | Meta-analysis | Excluded | Non-randomized controlled trial |
| 16 | Johnson et al.2017[16] | Review | Excluded | Non-randomized controlled trial |
| 17 | Tu et al. 2024[17] | Randomized Controlled Trial | Excluded | Insufficient data for outcome |
| 18 | Yuan et al. 2014[18] | Randomized Controlled Trial | Excluded | Insufficient data for outcome |
| 19 | Lang et al.2007[19] | Randomized Controlled Trial | Excluded | Insufficient data for outcome |
| 20 | Itoh et al.2008[20] | Randomized Controlled Trial | Excluded | Insufficient data for outcome |
| 21 | Wang H et al.[21] | Randomized Controlled Trial | Excluded | Insufficient data for outcome |

**Reference**

1. Sun K, Wang L, Hu X, Wang G. Effects of early transcutaneous acupoint electrical stimulation on postoperative cognition of elderly patients with hip fractures(in Chinese). Chinese Journal of Medical Physics. 2018;35(9):1075-9. doi: 10.3969/j.issn.1005-202X.2018.09.015.

2. Yang H, Zhong B, Zhong W. The Effects of Transcutaneous Electrical Acupoint Stimulation on Postoperative Delirium and Biomarkers in Elderly Patients with Hip Fracture (in Chinese). Modern Diagnosis and Treatment. 2023;34(15):2272-4.

3. Lan F, Xu N, Wang T. Study on Transcutaneous Electrical Acupoints Stimulation for Early postoperative pain relief after total hip arthroplasty in elderly patients (in Chinese). Beijing Medicine. 2017;39(10):1003-6.

4. Sun P, Peng L, Huizhou L, Mingjie W, Shuang Z, Zhao L, et al. Effect of percutaneous acupoint electrical stimulation on patient-controlled intravenous analgesia in elderly patients after total knee arthroplasty (in Chinese). Journal of Clinical Anesthesiology. 2019;35(3):243-6.

5. Duan C, Cui Z, Zhang X, Wen R, Li X. Effect of transcutaneous electrical acupoint stimulation on postoperative cognitive dysfunction in elderly patients (in Chinese). Hainan Medicine. 2019;30(16):2071-4. doi: 10.3969/j.issn.1003-6350.2019.16.010.

6. Yin Z, Meng Z, Lin S, Gao J, Chen X. Impacts of electrical acupoint stimulation on postoperative cognitive dysfunction and inflammation cytokines in elderly patients (in Chinese). Chinese Journal of Acupuncture and Moxibustion. 2015;4(4):159-63. doi: 10.3877/cma.j.issn.2095-3240.2015.04.001.

7. Lu B, Ding L, Wang L, Ke H, Yu B. Effect of Transcutaneous Electrical Acupoint Stimulation on Cerebral Oxygen Metabolism, Postoperative Cognitive Function and Analgesic Effect in Elderly Patients Undergoing Hip Replacement (in Chinese). Progress in Modern Biomedicine. 2019;19(16):3094-7. doi: 10.13241/j.cnki.pmb.2019.16.017.

8. Liu Y, Li Y, Cao X, Qi Y, Zhang Y. Effect of transcutaneous electrical acupuncture stimulation on postoperativecognitive dysfunction in elderly patients undergoing controlled hypotension (in Chinese). Journal of Xinjiang Medical University. 2019;42(3):406-9. doi: 10.3969/i.issn.1009-5551.2019.03.029.

9. Peng Y, Qiu Y, Chen H, Bai Y, Liu J, Liu Z, et al. Effects of perioperative transcutaneous electrical acupoint stimulation on intravenous patient-controlled analgesia in elderly patients after total hip arthroplasty (in Chinese). Hebei Medical Journal. 2019;41(19):2972-5. doi: 10.3969/j.issn.1002-7386.2019.19.024.

10. Wang D, Peng C, Ma T, Hu J. Effect of Transcutaneous Acupoint Electrical Stimulation on Postoperative Cognitive Function in Elderly Patients with Artificial Femoral Head Replacement (in Chinese). Chinese Archives of Traditional Chinese Medicine. 2016;34(2):431-3. doi: 10.13193 /j.issn.1673-7717.2016.02.052.

11. Wang J, Zhang W, Liu S, Pang J. Observation of the Analgesic Effects of Transcutaneous Electrical Acupoint Stimulation after Total Hip Arthroplasty (in Chinese). Shaanxi Journal of Traditional Chinese Medicine. 2017;38(7):962-4. doi: 10.3969/i.issn.1000-7369.2017.07.076.

12. Ge Y. Observation of the Analgesic Effect of Transcutaneous Acupoint Electrical Stimulation During the Perioperative Period of Total Hip Arthroplasty(in Chinese). Zhejiang Journal of Traditional Chinese Medicine. 2023;58(6):439. doi: 10.13633/j.cnki.zjtcm.2023.06.010.

13. Li W, Wang B, Wang K, Xu M, Yin H, He X. Effect of transcutaneous electrical nerve stimulation on pain after total hip arthroplasty(in Chinese). Chin J Bone Joint Surg. 2020;13(3). doi: 10.3969/j.issn.2095-9958.2020.03.13.

14. Tan S-Y, Jiang H, Ma Q, Ye X, Fu X, Ren Y-F, et al. Effects of transcutaneous electrical acupoint stimulation on early postoperative pain and recovery: a comprehensive systematic review and meta-analysis of randomized controlled trials. Frontiers in Medicine. 2024;11:1302057.

15. Guo F, Yan Y, Sun L, Han R, Zheng L, Qin Y, et al. Transcutaneous electrical acupoint stimulation for preventing postoperative delirium: a meta-analysis. Neuropsychiatric Disease and Treatment. 2023:907-20.

16. Johnson MI. Transcutaneous electrical nerve stimulation (TENS) as an adjunct for pain management in perioperative settings: a critical review. Expert review of neurotherapeutics. 2017;17(10):1013-27.

17. Tu Y, Qu S, Lin S, Gu S, Gao J. Effect of transcutaneous electrical acupoint stimulation on postoperative urinary function in elderly patients undergoing total hip arthroplasty. Zhongguo Zhen Jiu. 2024;44(4):395-9. doi: 10.13703/j.0255-2930.20230302-k0005.

18. Yuan L, Tang W, Wang J, Fu GQ. Effects of transcutaneous electrical acupoint stimulation combined with general anesthesia on cerebral oxygen metabolism in elderly hip replacement patients during controlled hypotension. Zhen Ci Yan Jiu. 2014;39(1):7-11, 9.

19. Lang T, Barker R, Steinlechner B, Gustorff B, Puskas T, Gore O, et al. TENS relieves acute posttraumatic hip pain during emergency transport. Journal of Trauma and Acute Care Surgery. 2007;62(1):184-8.

20. Itoh K, Hirota S, Katsumi Y, Ochi H, Kitakoji H. A pilot study on using acupuncture and transcutaneous electrical nerve stimulation (TENS) to treat knee osteoarthritis (OA). Chinese medicine. 2008;3:1-5.

21. Wang H, Xie Y, Zhang Q, Xu N, Zhong H, Dong H, et al. Transcutaneous electric acupoint stimulation reduces intra-operative remifentanil consumption and alleviates postoperative side-effects in patients undergoing sinusotomy: a prospective, randomized, placebo-controlled trial. BJA: British Journal of Anaesthesia. 2014;112(6):1075-82. doi: 10.1093/bja/aeu001.
