# Supplementary material for: Evaluation of transcutaneous electrical acupoint stimulation for improving pain and cognitive function in elderly patients around the perioperative period of hip replacement surgery: A meta-analysis
Source: PLoS One. 2024 Oct 21;19(10):e0309673. doi: 10.1371/journal.pone.0309673 (PMC11493289; doi:10.1371/journal.pone.0309673)
Supplement: S2 File — (DOCX) [file pone.0309673.s003.docx]

| Author & Year | Random Sequence Generation | Allocation Concealment | Blinding of Participants and Personnel | Blinding of Outcome Assessment | Incomplete Outcome Data | Selective Reporting | Other Bias |
| --- | --- | --- | --- | --- | --- | --- | --- |
| Duan et al.2019 | U | U | U | U | U | U | U |
| Ge et al.2023 | L | U | U | U | L | L | L |
| Lan et al.2017 | L | L | H | H | L | L | L |
| Li et al.2020 | L | U | L | U | L | L | L |
| Liu et al.2019 | L | L | L | L | L | U | U |
| Peng et al.2019 | L | L | L | L | L | L | L |
| Sun K et al.2018 | L | L | U | U | L | L | L |
| Sun PH et al.2019 | L | L | L | L | L | L | L |
| Wang DD et al.2016 | L | U | L | U | U | U | U |
| Wang JW et al.2017 | L | L | U | U | U | U | U |
| Yang et al.2023 | L | L | L | U | L | L | L |
| Yin et al. 2015 | L | U | U | U | L | L | L |

U unclear risk; L low risk; H high risk.

Criteria for Each Domain:

1.Random Sequence Generation:

Low Risk: Methods such as random number tables, coin toss, dice roll, computer-generated random numbers, or drawing lots.

High Risk: Quasi-random methods, e.g., allocation based on admission date, birth date, or patient ID number; allocation by personal choice or availability of test results.

Unclear Risk: Mentions "random allocation" or "random" without specific description.

2.Allocation Concealment:

Low Risk: Neither the investigator nor the participants know the upcoming allocation, using techniques like central randomization, coded containers, sequentially numbered, sealed, opaque envelopes, or on-site computer randomization.

High Risk: The investigator or participants are aware of the allocation, e.g., using alternation, or allocation based on date of birth, admission number; unsealed or transparent envelopes.

Unclear Risk: Mentions "random" or "random allocation" but no details on concealment.

3.Blinding of Participants and Personnel:

Low Risk: Blinding applied to main researchers and participants, or no blinding but outcome unlikely to be influenced by lack of it.

High Risk: No blinding.

Unclear Risk: Insufficient information; blinding not mentioned or unclear if it was adequate.

4.Blinding of Outcome Assessment:

Low Risk: Blinding applied to outcome assessors, or no blinding but outcome unlikely to be influenced by lack of it.

High Risk: No blinding.

Unclear Risk: Insufficient information; blinding not mentioned or unclear if it was adequate.

5.Incomplete Outcome Data:

Low Risk: No missing data, or missing data does not significantly affect the results (e.g., few missing patients).

High Risk: Significant missing data, likely to impact effect size (results inconsistent with most similar studies); discrepancies between Per Protocol (PP) and Intention to Treat (ITT) analysis.

Unclear Risk: Incomplete data, or description of data completeness is missing or difficult to assess.

6.Selective Reporting:

Low Risk: Protocol available, all pre-specified outcomes reported. No protocol but all major outcomes reported.

High Risk: Pre-specified major outcomes not fully reported, or outcome measurement/analysis methods not specified in the registered protocol.

Unclear Risk: Insufficient information, difficult to judge if selective reporting is present.

7.Other Sources of Bias:

Low Risk: No significant sources of bias identified in the included studies.

High Risk: Presence of one or more significant biases, e.g., significant baseline imbalances, early study termination.

Unclear Risk: Insufficient information, unclear whether identified issues lead to bias.
