# Supplementary material for: Evaluation of transcutaneous electrical acupoint stimulation for improving pain and cognitive function in elderly patients around the perioperative period of hip replacement surgery: A meta-analysis
Source: PLoS One. 2024 Oct 21;19(10):e0309673. doi: 10.1371/journal.pone.0309673 (PMC11493289; doi:10.1371/journal.pone.0309673)
Supplement: Attachment — Submitted filename: Response to Reviewers.docx [file pone.0309673.s005.docx]

**List of Responses**

Dear Editor and Reviewers:

Thank you for your letter and for the reviewers’ comments concerning our manuscript entitled “Evaluation of Transcutaneous Electrical Acupoint Stimulation for Improving Pain and Cognitive Function in Patients Around the Perioperative Period of Hip Replacement Surgery: A Meta-Analysis”. ID:PONE-D-24-21132. Those comments are all valuable and very helpful for revising and improving our paper, as well as the important guiding significance to our research. We have studied comments carefully and have made correction which we hope meet with approval. Revised portion are marked in red in the revised paper. The main corrections in the paper and the responds to the reviewer’s comments are as flowing:

Responds to the reviewer’s comments:

Reviewer #1:

Comment 1: The manuscript with title "Evaluation of Transcutaneous Electrical Acupoint Stimulation for Improving Pain and Cognitive Function in Patients Around the Perioperative Period of Hip Replacement Surgery: A Meta-Analysis" was good written and designed.

Answer: Thank you for your approval.

Reviewer #2:

Comment 1: In this manuscript, the research team employed meta-analysis and systematic evaluation to investigate the efficacy and safety of transcutaneous electrical acupoint stimulation in patients around the perioperative period of hip replacement surgery. The author's discussion is clearly expressed, allowing readers to easily comprehend the study's theme.

Answer: Thank you for your approval.

Comment 2: Due to the small number of documents included in this study, it is recommended to consider expanding the age range of the subjects. If elderly patients undergoing hip replacement surgery are selected, please describe the purpose of the study in the title and the full text.

Answer: We would like to express our sincere gratitude for your valuable feedback and insightful comments.

Regarding your suggestion to expand the age range of the subjects, we appreciate your concern. However, the primary population undergoing hip replacement surgery is indeed elderly patients. Our study was specifically aimed at providing clinical guidance for this patient group. We apologize for not emphasizing this focus on elderly patients earlier in the manuscript.

In response to your suggestion, we have made the necessary revisions. We have updated the title to "Evaluation of Transcutaneous Electrical Acupoint Stimulation for Improving Pain and Cognitive Function in Elderly Patients Around the Perioperative Period of Hip Replacement Surgery: A Meta-Analysis" to better reflect the targeted population. Additionally, we have emphasized the focus on elderly patients throughout the full text, including in the purpose.

All changes have been highlighted in red in the revised manuscript for your convenience

Comment 3: Based on Table 2, it is evident that China is predominantly involved in research within this field. Therefore, it is essential to search four renowned databases in China to ensure comprehensive retrieval of literature. Additionally, please update the retrieval date.

Answer: We sincerely appreciate your insightful comments and valuable suggestions.

In response to your recommendation, we have conducted a more thorough literature search by adding two additional Chinese databases, the VIP Database and SinoMed. We have re-screened all four Chinese databases to ensure comprehensive retrieval of relevant literature. The complete search strategies have been included in Table 1 for your reference.

As a result of this extended search, we identified two new studies, Ge et al.[1] and Li et al.[2], which have been included in our analysis. We have updated the data, figures, tables, and the retrieval date accordingly.

1.	Ge Y. Observation of the Analgesic Effect of Transcutaneous Acupoint Electrical Stimulation During the Perioperative Period of Total Hip Arthroplasty(in Chinese). Zhejiang Journal of Traditional Chinese Medicine. 2023;58(6):439. doi: 10.13633/j.cnki.zjtcm.2023.06.010.

2.	Li W, Wang B, Wang K, Xu M, Yin H, He X. Effect of transcutaneous electrical nerve stimulation on pain after total hip arthroplasty(in Chinese). Chin J Bone Joint Surg. 2020;13(3). doi: 10.3969/j.issn.2095-9958.2020.03.13.

Comment 4: The author should consider whether there are different measurement methods or units between VAS and MMSE, which will bring heterogeneity. Therefore, is it more accurate to utilize SMD in the scoring table?

Answer: Thank you for your thoughtful feedback and for raising an important point regarding potential heterogeneity due to different measurement methods or units between VAS and MMSE.

We agree that this could indeed introduce heterogeneity in our analysis. Following your suggestion, we have utilized the Standardized Mean Difference (SMD) for the following four indicators: (1) Postoperative Day 1 Visual Analog Scale, (2) Postoperative Day 2 Visual Analog Scale, (3) Postoperative Day 1 Mini-Mental State Examination, and (4) Postoperative Day 3 Mini-Mental State Examination. We have updated the forest plots accordingly.(Please See Fig 3 and Fig 4)

Comment 5: After evaluating the risk bias of the included literature, two graphs are usually generated. Please fill in the missing detail evaluation chart so that readers can understand the risk of bias in each included study.

Answer: We sincerely appreciate your careful evaluation and constructive suggestion.

In response to your comment, we have replaced the previous quality assessment figures with detailed evaluation charts. These charts now clearly present the risk of bias for each included study, including the two newly added articles. This will allow readers to better understand the risk of bias in each study.(Please See Fig 2)

Comment 6: Publication bias is usually carried out when an outcome indicator meets the number of 10 or more studies.

Answer: Thank you for your insightful comment regarding the assessment of publication bias.

After reviewing the Cochrane Handbook, we agree with your observation. As a result, we have removed the publication bias assessment and the associated funnel plots from our analysis to avoid presenting potentially misleading or non-objective results.

Comment 7: In the results section, please add the P value of each comparison result.

Answer: Thank you for your attention to detail and for your valuable feedback.

In response to your suggestion, we have added the P values for each comparison result in the results section of the manuscript. These additions will provide a clearer understanding of the statistical significance of our findings.

We tried our best to improve the manuscript and made some changes in the manuscript. These changes will not influence the content and framework of the paper. And all the changes have mark in red in the revised paper. We appreciate for Editors/Reviewers’ warm work earnestly, and hope that the correction will meet with approval.

Once again, thank you very much for your comments and suggestions.
